# Sclerotome-derived PDGF signaling functions as a niche cue responsible for primitive erythropoiesis

**DOI:** 10.1242/dev.201807

**Published:** 2023-11-16

**Authors:** Aihua Mao, Zhuyun Li, Guozhu Ning, Zhengrong Zhou, Chiju Wei, Jianchao Li, Xinyu He, Qiang Wang

**Affiliations:** ^1^ Guangdong Provincial Key Laboratory of Marine Biotechnology, Institute of Marine Sciences, Shantou University, Shantou, Guangdong 515063, China; ^2^ Innovation Centre of Ministry of Education for Development and Diseases, Sixth Affiliated Hospital, School of Medicine, South China University of Technology, Guangzhou 510006, China; ^3^ School of Life Sciences, University of Science and Technology of China, Hefei, Anhui 230026, China; ^4^ Affiliated Hospital of Guangdong Medical University and Key Laboratory of Zebrafish Model for Development and Disease, Guangdong Medical University, Zhanjiang 524001, China; ^5^ State Key Laboratory of Molecular Developmental Biology, Institute of Genetics and Developmental Biology, Chinese Academy of Sciences, Beijing 100101, China

**Keywords:** Primitive erythropoiesis, PDGF, Sclerotome, ERK1/2, Cell differentiation

## Abstract

Primitive erythropoiesis serves a vital role in embryonic development, generating primitive red blood cells responsible for transportation of oxygen throughout the body. Although diverse niche factors are known to function in definitive hematopoiesis, the microenvironment contributing to primitive hematopoiesis remains largely elusive. Here, we report that platelet-derived growth factor (PDGF) signaling is required for erythroid progenitor differentiation in zebrafish. Ablating *pdgfαa* (also known as *pdgfaa*) and *pdgfαb* (also known as *pdgfab*) or blocking PDGF signaling with an inhibitor impairs erythroid progenitor differentiation, thus resulting in a significant decrease in the number of erythrocytes. We reveal that *pdgfαb* is expressed in sclerotomal cells, and that its receptor genes, *pdgfra* and *pdgfrb*, are expressed in the adjacent erythroid progenitor cells. Sclerotome-specific overexpression of *pdgfαb* effectively restores primitive erythropoiesis in *pdgfαa^−/−^;pdgfαb^−/−^* mutant embryos. In addition, we have defined ERK1/2 signaling as a downstream pathway of PDGF signaling during embryonic erythropoiesis. Taken together, our findings indicate that PDGF signaling derived from sclerotome functions as a niche cue for primitive erythropoiesis.

## Teaser

**Summary:** Activation of platelet-derived growth factor signaling in sclerotome microenviroment facilitates zebrafish primitive erythropoiesis.

## INTRODUCTION

Erythrocytes are the most common cell type in blood and are responsible for oxygen transportation and vascular morphogenesis during embryo development (Baron et al., 2012; Zhou et al., 2021). Erythrocytes arise in two successive waves of hematopoiesis, primitive and definitive. As the hematopoietic processes and molecular mechanisms in zebrafish are highly conserved in mammals, zebrafish has been regarded as a powerful model for the study of hematopoiesis (de Jong and Zon, 2005; Jagannathan-Bogdan and Zon, 2013). In mammals, the early hematopoietic wave takes place in the yolk sac, where the primitive erythrocytes are generated. In zebrafish, embryonic hematopoiesis originates from the intermediate cell mass (ICM) of the posterior lateral plate mesoderm and the rostral blood island located in the anterior lateral plate mesoderm (Davidson and Zon, 2004; Ellett and Lieschke, 2010). The second wave, known as definitive hematopoiesis, gives rise to the hematopoietic stem cells (HSCs), which facilitate lifelong replenishment of all major hematopoietic lineages. HSCs emerge from the aorta-gonad-mesonephros region in mammals and ultimately migrate to the mammalian fetal liver and the bone marrow, which function as niches for their proliferation and differentiation. This is equivalent to the HSC transition from the ventral wall of the dorsal aorta (DA) to the kidney marrow in zebrafish (Bertrand et al., 2010). The definitive hematopoietic niches serve important roles to maintain the self-renewal capacity of HSCs, protect them from exhaustion and regulate their differentiation, which is crucial for blood system homeostasis. These dedicated niches have been widely investigated in recent decades and comprise vascular endothelial cells and mesenchymal stromal cells, along with diverse molecular signals (Calvi et al., 2003; Morrison and Scadden, 2014; Rafii et al., 2015; Touret et al., 2022). Conversely, little is known about the components of the primitive hematopoietic niche and how they work in concert to regulate embryonic erythropoiesis.

The platelet-derived growth factor (PDGF) family comprises four different gene products (PDGFA, PDGFB, PDGFC and PDGFD), which exist in disulfide-linked dimeric forms. There are two receptors for PDGFs, PDGFRα and PDGFRβ (Pdgfra and Pdgfrb, respectively, in zebrafish) (Kazlauskas, 2017; Shim et al., 2010). Binding of PDGF ligands to PDGF receptors (PDGFRs) induces receptor dimerization, autophosphorylation on tyrosine residues and subsequent activation of downstream signaling cascades. PDGF signaling is one of the pivotal signaling pathways regulating cell proliferation, migration and differentiation. It has been demonstrated to promote the expansion of several hematopoietic cell types *ex vivo* (Su et al., 2001, 2002). In addition, aberrant activation of PDGF signaling is associated with hematopoietic malignancies, such as myeloid neoplasms with hypereosinophilia and large granular lymphocytic leukemia (Demoulin and Montano-Almendras, 2012; Yang et al., 2010). These findings indicate that PDGF signaling has a role in physiological and pathological hematopoietic processes. Interestingly, PDGF signaling has been recently reported to contribute to the definitive hematopoietic niche (da Bandeira et al., 2022; Damm and Clements, 2017). However, it is unknown whether PDGF signaling functions in primitive hematopoiesis. Here, we identify that PDGF signaling derived from the sclerotome acts as a niche cue that plays a role in embryonic erythropoiesis, facilitating the differentiation of erythroid progenitors and the formation of primitive red blood cells (RBCs).

## RESULTS AND DISCUSSION

### Ablation of *pdgfαa* and *pdgfαb* impairs primitive erythropoiesis

**Fig. 1.**
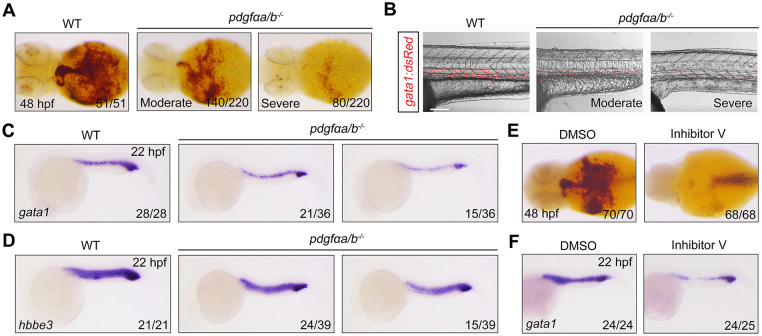
**Suppression of PDGF signaling leads to a reduction of erythrocytes.** (A) Detection of hemoglobin levels by *o*-dianisidine staining in wild-type (WT) and *pdgfαa^−/−^*;*pdgfαb^−/−^* (*pdgfαa/b^−/−^*) embryos at 48 hpf. Ventral view. (B) Merged brightfield and confocal images from live imaging of *pdgfαa^−/−^*;*pdgfαb^−/−^* embryos in the *Tg(gata1:dsRed)* background at 32 hpf. Lateral view with anterior to the left. Scale bar: 100 μm. (C,D) Expression analysis of (C) *gata1* and (D) *hbbe3* in WT and *pdgfαa^−/−^*;*pdgfαb^−/−^* embryos at 22 hpf by *in situ* hybridization. Lateral view with anterior to the left. (E,F) WT embryos were treated with 0.25 μM inhibitor V from shield stage then harvested at the indicated developmental stages for (E) *o*-dianisidine staining (ventral view) or (F) *gata1 in situ* hybridization (lateral view with anterior to the left). Images shown are representative of the observed phenotypes. In A and C-F, the number of embryos displaying each phenotype out of the total number assayed is indicated. Images in B are representative of 35 WT embryos and 40 *pdgfαa/b*^−/−^ embryos. The number of red blood cells was moderately decreased in about two-thirds of the *pdgfαa/b*^−/−^ embryos, and severely reduced in the rest of the mutant embryos.

In a previous study, we generated double mutants lacking *pdgfαa* (also known as *pdgfaa*) and *pdgfαb* (also known as *pdgfab*), the zebrafish orthologs of *PDGFA*, and found that the *pdgfαa^−/−^;pdgfαb^−/−^* double mutants have malformations of the pharyngal arch artery (Mao et al., 2019). Intriguingly, under brightfield microscopy, we observed that there appeared to be significantly fewer blood cells in the *pdgfαa^−/−^;pdgfαb^−/−^* embryos. To confirm this observation, *o*-dianisidine staining was applied at 48 h post fertilization (hpf) to detect the formation of RBCs by evaluating hemoglobin levels. Indeed, an obvious decrease in hemoglobin expression was found in *pdgfαa^−/−^;pdgfαb^−/−^* mutants (Fig. 1A), suggesting an important role of *pdgfαa* and *pdgfαb* in blood cell development. To further monitor erythroid populations, we crossed the *Tg(gata1:dsRed)* strain with *pdgfαa^−/−^;pdgfαb^−/−^* fish*.* A dramatic reduction in the number of circulating RBCs was observed in the mutants (Fig. 1B; Movie 1), whereas no obvious abnormalities of heart rhythm or contraction occurred. After deletion of *pdgfαa* and *pdgfαb*, the expression of the erythrocyte-specific markers *gata1a* (named *gata1* for short) and *hbbe3* was slightly decreased in ∼60% of embryos and markedly reduced in ∼40% of embryos at 22 hpf (Fig. 1C,D). These results imply that PDGF signaling is involved in primitive erythropoiesis.

Next, wild-type embryos were treated with inhibitor V, a selective PDGFR tyrosine kinase inhibitor, from the shield stage to the long-pec stage. As expected, a severe decrease in the number of RBCs was detected in the resulting embryos, as revealed by *o*-dianisidine staining (Fig. 1E). Decreased expression of *gata1* confirmed the defective erythropoiesis in animals treated with inhibitor V (Fig. 1F).

To determine whether *pdgfαa* and *pdgfαb* are both involved in hematopoiesis, we examined the expression of *gata1* and *hbbe3* in *pdgfαa^−/−^* or *pdgfαb^−/−^* mutant embryos using *in situ* hybridization. No obvious changes were observed in primitive hematopoiesis at 14 hpf and 22 hpf (Fig. S1). This might be due to compensatory effects of *pdgfαa* and *pdgfαb*, as we have discussed in previous work (Mao et al., 2019). Collectively, these results indicate that both *pdgfαa* and *pdgfαb* play a crucial function in primitive erythropoiesis.

HSCs, a unique cell population arising in definitive hematopoiesis, are capable of self-renewal and multilineage differentiation. We next investigated HSC formation in *pdgfαa^−/−^;pdgfαb^−/−^* embryos so as to define the role of these genes in definitive hematopoiesis. Expression of the HSC markers *runx1* and *cmyb* (also known as *myb*) was unaffected in *pdgfαa^−/−^;pdgfαb^−/−^* mutants (Fig. S2A,B). In addition, the thymus epithelial cell marker *ccl25a* and T lymphocyte marker *rag1* were expressed normally in the double mutants (Fig. S2C,D). Taken together, these results suggest that *pdgfαa* and *pdgfαb* are dispensable for definitive hematopoiesis. However, PDGF signaling has been reported to be necessary for HSC specification by guiding the migration of neural crest cells to the DA, and HSCs have been found to be significantly reduced in *pdgfra*-deficient embryos (Damm and Clements, 2017). Interestingly, in our study, the expression pattern of the neural crest marker *crestin* remained unchanged upon depletion of both *pdgfαa* and *pdgfαb* (Fig. S2E). This could be explained by the existence of other PDGF ligands that activate PDGFRs during HSC formation (Andrae et al., 2008; Wiens et al., 2010).

### *pdgfαa* and *pdgfαb* are indispensable for erythrocyte progenitor differentiation

The dramatic decrease in the number of RBCs in *pdgfαa^−/−^;pdgfαb^−/−^* double mutants raised the possibility that *pdgfαa* and *pdgfαb* inhibit erythroid progenitor proliferation or accelerate their apoptosis. To test this possibility, terminal deoxynucleotidyl transferase-mediated dUTP nick end labeling (TUNEL) and bromodeoxyuridine (BrdU) incorporation assays were performed. TUNEL staining revealed no obvious apoptotic signal in the erythrocyte progenitor cells of wild-type and *pdgfαa^−/−^;pdgfαb^−/−^* embryos (Fig. S3A,B). Meanwhile, the BrdU incorporation assays showed no apparent difference in erythroid progenitor proliferation between groups (Fig. S3C,D). Furthermore, similar results were observed in embryos treated with inhibitor V (Fig. S4). These data indicate that loss of *pdgfαa and pdgfαb* does not affect the proliferation and survival of erythrocyte progenitor cells.

**Fig. 2.**
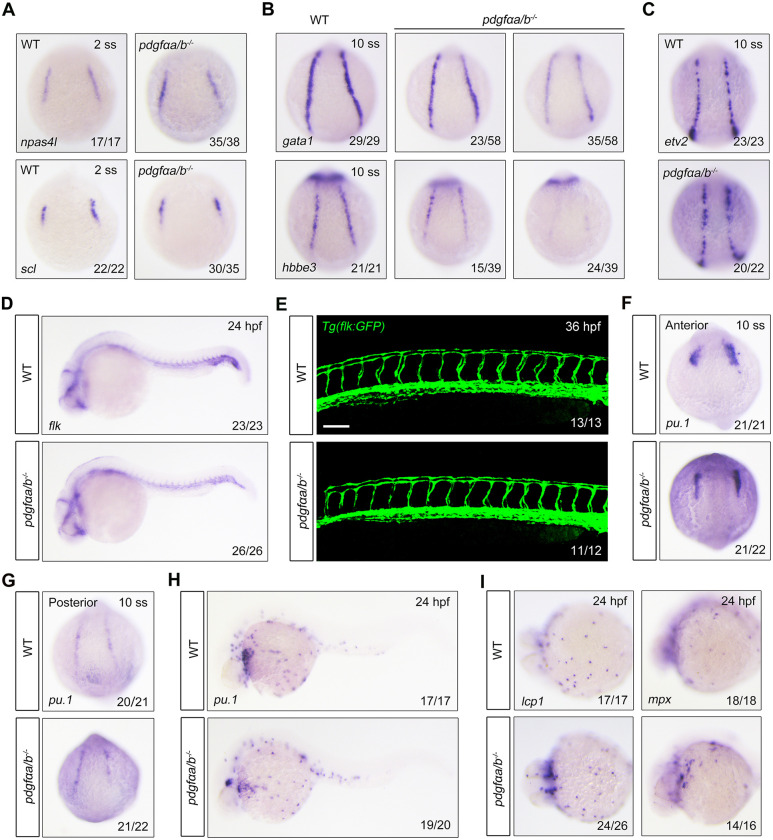
***pdgfαa* and *pdgfαb* are necessary for erythroid progenitor differentiation but are dispensable for the development of endothelial and myeloid lineages.** (A) Expression patterns of *npas4 l* and *scl* in wild-type (WT) and *pdgfαa^−/−^*;*pdgfαb^−/−^* (*pdgfαa/b^−/−^*) embryos at the two-somite stage (2 ss) as revealed by *in situ* hybridization. Dorsal view. (B) Expression of *gata1* and *hbbe3* in WT and *pdgfαa^−/−^*;*pdgfαb^−/−^* embryos at the 10-somite stage (10 ss) assayed by *in situ* hybridization. Dorsal view. (C,D) *In situ* hybridization expression analysis of (C) *etv2* (dorsal view) and (D) *flk* (lateral view with head to the left) in WT and *pdgfαa^−/−^*;*pdgfαb^−/−^* embryos at the indicated stages. (E) Confocal images of *pdgfαa^−/−^*;*pdgfαb^−/−^* mutants in the *Tg(flk:GFP)* background at 36 hpf. Lateral view. Scale bar: 50 μm. (F-H) Expression patterns of *pu.1* in WT and *pdgfαa^−/−^*;*pdgfαb^−/−^* embryos at the indicated stages as revealed by *in situ* hybridization. Dorsal view. (I) Expression analysis of *lcp1* and *mpx* in WT and *pdgfαa^−/−^*;*pdgfαb^−/−^* embryos at 24 hpf by *in situ* hybridization. Ventral view in the left panel and lateral view in the right panel. Images shown are representative of the observed phenotypes. The number of embryos displaying each phenotype out of the total number assayed is indicated.

We then sought to examine the emergence of erythrocyte progenitor cells in *pdgfαa^−/−^;pdgfαb^−/−^* mutants. Because hemangioblasts arise out of the ventral mesoderm and differentiate into hematopoietic cells and vascular endothelial cells (Vogeli et al., 2006), we first evaluated the expression of ventral mesodermal genes *eve1* and *vent* and found that ablation of *pdgfαa* and *pdgfαb* did not affect the formation of ventral mesoderm (Fig. S5). Moreover, expression analysis of the hemangioblast markers *npas4l* and *scl* (also known as *tal1*) in wild-type and mutant embryos at the two-somite stage revealed a comparable hemangioblast population (Fig. 2A). However, expression of the erythroid progenitor markers *gata1* and *hbbe3* was reduced in *pdgfαa^−/−^;pdgfαb^−/−^* mutants at the 10-somite stage (Fig. 2B), indicating impaired differentiation of hemangioblasts into erythroid progenitors. In contrast, we observed no significant difference in expression of *etv2* between the *pdgfαa^−/−^;pdgfαb^−/−^* mutants and the wild-type control at the 10-somite stage, suggesting that the development of endothelial progenitors is not affected by deletion of *pdgfαa* and *pdgfαb* (Fig. 2C). Moreover, no apparent vascular defect was observed in *pdgfαa^−/−^;pdgfαb^−/−^* embryos at later developmental stages (Fig. 2D,E). Consistent with the above observations, suppressing PDGF signaling with inhibitor V did not alter the formation of hemangioblast and the development of endothelial progenitors, but severely reduced the emergence of erythrocyte progenitors (Fig. S6).

Because primitive hematopoiesis consists of erythropoiesis and myelopoiesis, we next asked whether the generation of myeloid cells was also impaired when both *pdgfαa* and *pdgfαb* were deleted. The expression profiles of *pu.1* (also known as *spi1b*), *l-plastin* (also known as *lcp1*) and *mpx* revealed that the myeloid cells, including macrophages and granulocytes, were fully developed in both wild-type and mutant embryos (Fig. 2F-I).

Together, these data suggest that *pdgfαa* and *pdgfαb* are specifically required for the differentiation of erythroid progenitors from hemangioblasts during primitive hematopoiesis, but are unnecessary for the development of endothelial and myeloid lineages.

### Sclerotome-derived PDGF signaling functions in primitive erythropoiesis

**Fig. 3.**
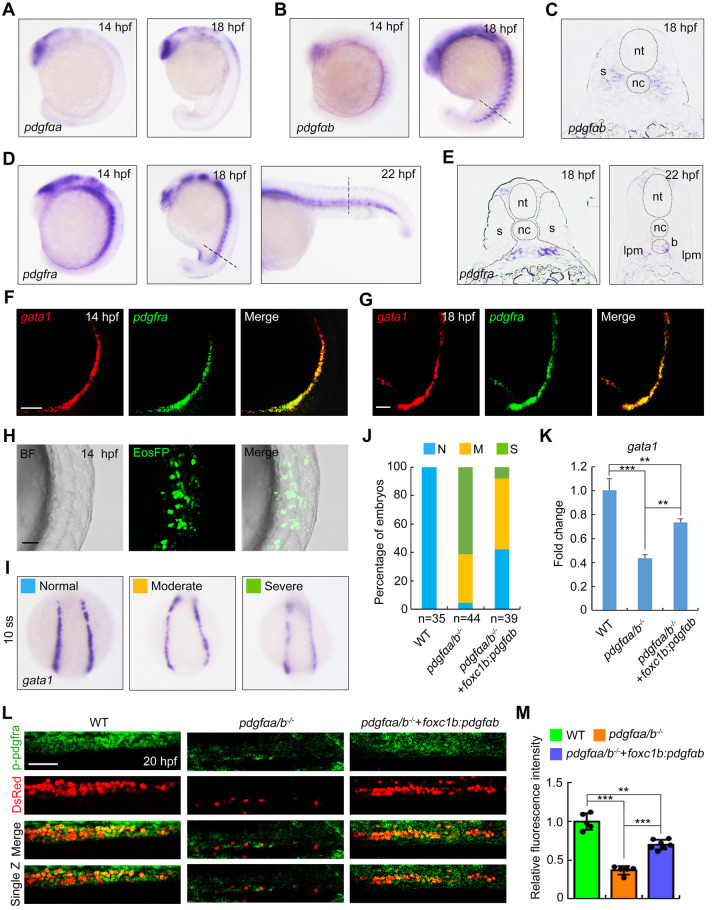
**Overexpression of *pdgfαb* in sclerotome is sufficient to rescue primitive erythropoietic defects in *pdgfαa^−/−^;pdgfαb^−/−^* embryos.** (A) Expression analysis of *pdgfαa* in wild-type embryos at the indicated stages by *in situ* hybridization. Lateral view with anterior to the top. (B,C) Expression profile of *pdgfαb* in wild-type embryos assayed by *in situ* hybridization at the indicated stages and displayed (B) as whole mounts (lateral view with anterior to the top) or (C) in section. Dashed line in B indicates the position of the section in C. (D,E) Expression patterns of *pdgfra* in wild-type embryos assayed by *in situ* hybridization at the indicated stages, shown as (D) whole mounts or (E) sections. Dashed lines in D represent the positions of the sections in E. B, blood cell; lpm, lateral plate mesoderm; nc, notochord; nt, neural tube; s, somite. (F,G) Colocalization analysis of *pdgfra* and *gata1* transcripts in wild-type embryos at (F) 14 hpf and (G) 18 hpf by dual-color fluorescence *in situ* hybridization. Lateral view with anterior to the top. Scale bars: 50 μm. (H-K) *pdgfαa^−/−^*;*pdgfαb^−/−^* (*pdgfαa/b^−/−^*) embryos were injected with 30 pg *foxc1b:pdgfαb-P2A-EosFP* plasmid and 200 pg Tol2 transposase mRNA at the one-cell stage, and then harvested for *in vivo* confocal imaging or *in situ* hybridization alongside uninjected WT and *pdgfαa/b*^−/−^ control embryos. (H) Representative brightfield (BF) and confocal images of a *pdgfαa/b*^−/−^ embryo injected with the plasmid *foxc1b: pdgfαb-P2A-EosFP* and Tol2 mRNA at 14 hpf. Lateral view with anterior to the top. Scale bar: 50 μm. (I) The transcripts of *gata1* were evaluated by *in situ* hybridization at 10-somite stage (10 ss). Representative images of the phenotypes observed in uninjected WT or *pdgfαa/b*^−/−^ control embryos and *pdgfαa/b*^−/−^ embryos injected with the indicated plasmid and Tol2 transposase mRNA. (J) The percentage of embryos scored as being normal (N) or as having moderate (M) or severe (S) defects for the indicated genotypes. (K) Quantitative real-time PCR was performed to examine the expression of *gata1* in the indicated embryos, and the values are expressed as mean±s.d. Fold change relative to WT from three independent experiments. One-way ANOVA with Scheffe post-hoc test was used to analyze the statistical differences between groups. **P<0.01, ***P<0.001. (L,M) Immunofluorescence assay of p-Pdgfra in *Tg(gata1:dsRed)* background embryos. WT and *pdgfαa/b*^−/−^ control embryos were uninjected, and *pdgfαa/b*^−/−^ embryos were injected with *foxc1b:pdgfαb-P2A-EosFP* plasmids and Tol2 transposase mRNA as described in H. (L) Lateral views with anterior to the left. Scale bar: 50 μm. (M) The average fluorescence intensity of p-Pdgfra in the ICM region of each group was calculated using ImageJ software, and the fluorescence intensity values relative to WT are shown as mean±s.d. (*n*>5) from three independent experiments. One-way ANOVA test with Scheffe post-hoc test was used to analyze the statistical differences between groups. **P<0.01, ***P<0.001.

Given the importance of *pdgfαa* and *pdgfαb* for early erythroid cell fate decisions, we asked whether the expression patterns of these ligands and their receptors are consistent with a role in primitive erythropoiesis. We first mapped the temporal-spatial distribution of *pdgfαa* and *pdgfαb* transcripts by performing whole-mount *in situ* hybridization (WISH) experiments. Expression of *pdgfαa* was observed in the central nervous system at 14 hpf and emerged in truncal epidermis at 18 hpf (Fig. 3A). Interestingly, *pdgfαb* transcript was enriched in the central nervous system and the ventral portion of the somite known as the sclerotome (Fig. 3B,C). On the other hand, the expression of *pdgfra*, the receptor gene of *pdgfαa* and *pdgfαb*, was mostly detected in the posterior lateral plate mesoderm at 14 hpf and then appeared in the ICM at later developmental stages (Fig. 3D,E). Furthermore, dual-color fluorescence *in situ* hybridization analysis showed that *pdgfra* was specifically expressed in the erythrocyte progenitor cells expressing *gata1* during somitogenesis (Fig. 3F,G). Such spatial-temporal proximity of ligand and receptor expression implies that PDGF signaling derived from sclerotomal cells might play an important role in primitive erythropoiesis.

In order to verify the above hypothesis, we conducted rescue experiments in *pdgfαa^−/−^;pdgfαb^−/−^* mutants by overexpression of *pdgfαb* under the control of the promoter of the sclerotome-specific gene *foxc1b* (Clements et al., 2011; Qiu et al., 2016). After injecting the *foxc1b:pdgfαb-P2A-EosFP* plasmid and Tol2 transposase mRNA into *pdgfαa^−/−^;pdgfαb^−/−^* embryos, robust green fluorescence was observed specifically in sclerotomal cells at 14 hpf (Fig. 3H). Concurrently, the decreased expression of *gata1* in *pdgfαa^−/−^;pdgfαb^−/−^* mutants was markedly recovered, as revealed by WISH and quantitative real-time PCR experiments (Fig. 3I-K).

Binding of PDGF ligands to their receptors triggers receptor dimerization and autophosphorylation on tyrosine residues. As expected, ablation of *pdgfαa* and *pdgfαb* significantly downregulated the phosphorylation level of PDGFRs in erythrocyte progenitors, as revealed by immunostaining experiments using an antibody that detected phosphorylated Pdgfra (p-Pdgfra), whose specificity was confirmed by western blotting analysis of inhibitor V-treated embryos (Fig. 3L,M; Fig. S7A). Importantly, the level of p-Pdgfra in *pdgfαa^−/−^;pdgfαb^−/−^* mutants was restored by overexpression of *pdgfαb* in the sclerotomal cells (Fig. 3L,M). Collectively, the above findings support the idea that PDGF signaling molecules from the sclerotome are essential for erythroid progenitor differentiation in the adjacent posterior lateral plate mesoderm.

It has been reported that *pdgfra* morpholino (MO)-injected embryos display normal primitive erythropoiesis (Damm and Clements, 2017). We further confirmed the efficiency of the MO that has been used to knockdown *pdgfra* expression (Damm and Clements, 2017). Indeed, injection of the *pdgfra* MO into wild-type embryos markedly decreased the level of p-Pdgfra and caused clear defects in the ventromedial migration of trunk neural crest cells (Fig. S7B,C). However, the expression of *gata1* in the ICM region of *pdgfra* morphants was not obviously changed (Fig. S7D,G). In general, PDGFR proteins form homo- or hetero-dimers to be activated by PDGF ligands (Kazlauskas, 2017; Shim et al., 2010). Interestingly, we found that *pdgfrb* was expressed in the hypochord and in the erythroid progenitor cells located near its ventral region (Fig. S7E), indicating that *pdgfrb* might be relevant to erythropoiesis. We then injected a previously validated antisense MO targeting *pdgfrb* into wild-type embryos (Wiens et al., 2010). We found that although *pdgfrb* single morphants showed no defect in primitive erythropoiesis, *pdgfra* and *pdgfrb* double morphants exhibited significantly reduced expression of *gata1* and *hbbe3* (Fig. S7F,G). These data indicate that *pdgfra* and *pdgfrb* function together to promote primitive erythropoiesis, similar to their role in regulating craniofacial development (Fantauzzo and Soriano, 2016).

### PDGF signaling regulates primitive erythropoiesis through a downstream ERK1/2 pathway

**Fig. 4.**
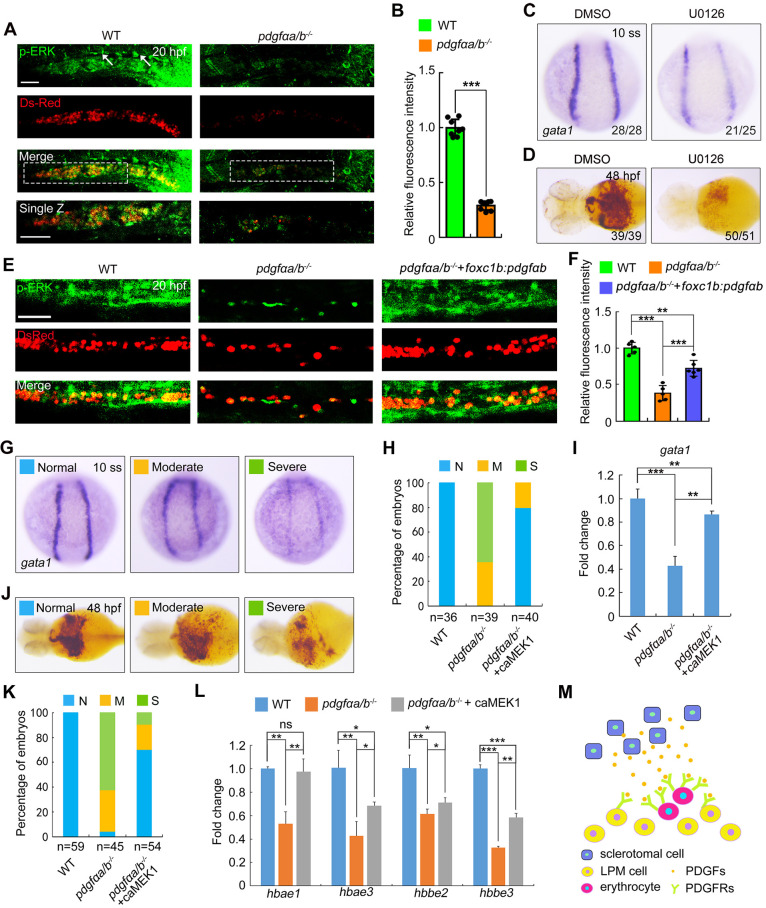
**ERK1/2 functions downstream of PDGF signaling to promote primitive erythrocyte development.** (A,B) Immunostaining of phosphorylated ERK1/2 (p-ERK) in wild-type (WT) and *pdgfαa^−/−^*;*pdgfαb^−/−^* (*pdgfαa/b^−/−^*) *Tg(gata1:dsRed)* embryos. (A) Representative confocal images. Single sections of the boxed areas are shown as enlarged images in the bottom panels. Lateral view with anterior to the left. The white arrows indicate endothelial cells of the DA. Scale bars: 50 μm. This experiment was repeated independently three times with similar results. (B) Average fluorescence intensity of p-ERK in the ICM region was calculated using ImageJ software, and the fluorescence intensity values relative to WT are shown as mean±s.d. (*n*=8). Two-tailed, unpaired Student's *t*-test was used to analyze the difference between groups. ***P<0.001. (C) *In situ* hybridization expression analysis of *gata1* in WT embryos treated with 25 μM U0126 or DMSO as a vehicle control from the shield stage to the 10-somite stage (10 ss). Dorsal view. (D) Determination of hemoglobin levels by *o*-dianisidine staining in U0126-treated WT embryos at 48 hpf. Ventral view. Images shown in C and D are representative of the observed phenotypes. The number of embryos displaying each phenotype out of the total number assayed is indicated. (E,F) Immunostaining staining of p-ERK at 20 hpf in *Tg(gata1:dsRed)* background embryos. *pdgfαa/b*^−/−^ embryos were injected with 30 pg *foxc1b-pdgfαb-P2A-EosFP* plasmids and 200 pg Tol2 transposase mRNA at the one-cell stage. WT and *pdgfaαa/b*^−/−^ control groups were uninjected. (E) Representative confocal images. Lateral views. Scale bar: 50 μm. (F) The average fluorescence intensity of p-ERK in the ICM region was calculated using ImageJ software, and the fluorescence intensity values relative to WT are shown as mean±s.d. (*n*≥5) from three independent experiments. One-way ANOVA with Scheffe post-hoc test was used to analyze the statistical differences between groups. **P<0.01, ***P<0.001. (G-I) *pdgfαa^−/−^*;*pdgfαb^−/−^* embryos were injected with 20 pg caMEK1 mRNA at the one-cell stage and then harvested alongside uninjected WT and *pdgfαa/b*^−/−^ control embryos for detection of *gata1* expression by *in situ* hybridization at 10 ss. (G) Representative images of observed phenotypes. Dorsal view. (H) The percentage of embryos scored as being normal (N) or as having moderate (M) or severe (S) defects for the indicated treatment groups. (I) Quantitative real-time PCR was performed to examine the expression of *gata1* in the indicated embryos, and the values are expressed as mean±s.d. Fold change relative to WT from three independent experiments. One-way ANOVA with Tukey post-hoc test to analyze the statistical differences between groups. **P<0.01, ***P<0.001. (J-L) Hemoglobin levels in embyros as described in G-I were examined at 48 hpf using *o*-dianisidine staining. (J) Representative images of phenotypes observed. Ventral view. (K) The percentage of embryos scored as being normal (N) or as having moderate (M) or severe (S) defects for the indicated treatment groups. (L) Quantitative real-time PCR was performed to examine the expression of *hbae1*, *hbae3*, *hbbe2* and *hbbe3*, and the values are expressed as mean±s.d. Fold change relative to WT from three independent experiments. One-way ANOVA with Tukey post-hoc test was used to analyze the statistical differences between groups. *P<0.05; **P<0.01; ***P<0.001; ns, not significant. (M) A proposed model illustrating that PDGF ligands derived from sclerotome interact with and activate their receptors in the nearby lateral plate mesoderm, thereby supporting erythroid progenitor differentiation. LPM, lateral plate mesoderm.

As PDGFRs have been reported to activate several downstream signaling cascades, including the AKT-PI3K and ERK1/2 pathways (Heldin, 2013; Hoch and Soriano, 2003), we sought to determine which pathway plays a primary role downstream of PDGFRs in primitive erythropoiesis. Firstly, phosphorylated AKT(Ser473) (p-AKT) and phospho-ERK1/2 (both Mapk3 and Mapk1 in zebrafish) were examined in *pdgfαa^−/−^;pdgfαb^−/−^* embryos. Immunostaining analysis revealed robust signals of phosphorylated AKT kinases in the ICM region of both wild-type and mutant embryos (Fig. S8). In contrast, the immunostaining signal of phosphorylated ERK1/2 in the ICM region was almost eliminated in *pdgfαa^−/−^;pdgfαb^−/−^* mutants as compared with that of control animals (Fig. 4A,B).

To verify the necessity of ERK1/2 signaling during erythrocyte development, the specific inhibitor U0126 was used to block the phosphorylation of ERK1/2 (Hawkins et al., 2008). Wild-type embryos treated with U0126 displayed drastically decreased expression of *gata1* at 14 hpf, and exhibited severe defects in erythropoiesis at 48 hpf, as indicated by *o*-dianisidine staining (Fig. 4C,D). Notably, specific overexpression *pdgfαb* in the sclerotome effectively restored the levels of phosphorylated ERK1/2 in the *pdgfαa^−/−^;pdgfαb^−/−^* mutants (Fig. 4E,F). Importantly, when a constitutively active form of Mek1 (caMEK1; Bolcome and Chan, 2010), the upstream activator of ERK1/2, was overexpressed in *pdgfαa^−/−^;pdgfαb^−/−^* embryos, the emergence of erythroid progenitors and the development of erythrocytes was restored (Fig. 4G-L). Taken together, these findings indicate that ERK1/2 signaling acts downstream of PDGF signaling to regulate primitive erythropoiesis.

Based on these results, we propose a model for the function of PDGF signaling in embryonic erythropoiesis (Fig. 4M). PDGF ligands derived from sclerotome interact with and activate their receptors in the nearby lateral plate mesoderm, subsequently activating the downstream ERK1/2 pathway to eventually support erythroid progenitor differentiation. Thus, our study highlights the crucial role of sclerotome-derived PDGF signaling as a primitive erythropoietic niche cue.

It has been reported that deficiency of PDGFB or PDGFRβ leads to multisystem disorders in mice, including hematopoietic defects such as anemia and thrombocytopenia (Levéen et al., 1994; Soriano, 1994). More specifically, PDGF signaling has been demonstrated to help the establishment of the definitive hematopoietic niche (da Bandeira et al., 2022; Damm and Clements, 2017). In our study, we found that knockout of both *pdgfαa* and *pdgfαb* in zebrafish caused a dramatic decrease in the number of embryonic RBCs in circulation, which could be attributed to the defective differentiation of erythropoietic progenitors. The expression of *pdgfαb* was enriched in sclerotome, and the receptor genes *pdgfra* and *pdgfrb* were expressed in the adjacent lateral plate mesoderm. Their spatial localization facilitates ligand-receptor binding and signal initiation, thereby activating downstream ERK1/2 signaling to promote erythroid progenitor differentiation. Indeed, overexpression of *pdgfαb* in the sclerotome could effectively restore RBC formation in *pdgfαa^−/−^;pdgfαb^−/−^* mutants. Our findings strongly imply that sclerotome-derived PDGF signaling serves as a niche cue responsible for primitive hematopoiesis. Interestingly, it has been suggested that PDGFB signaling in the placental microenvironment plays a role in protecting hematopoietic stem/progenitor cells from definitive erythroid differentiation (Chhabra et al., 2012), implying that PDGF signaling might have distinct roles in primitive and definitive erythropoiesis.

## MATERIALS AND METHODS

### Zebrafish lines

The following zebrafish (*Danio rerio*) lines were used: wild type (Tübingen), *Tg(gata1:dsRed)*, *Tg(flk:GFP)*, *pdgfαa^−/−^*, *pdgfαb^−/−^* and *pdgfαa^−/−^*;*pdgfαb^−/−^* (Mao et al., 2019). Zebrafish embryos collected from controlled pair- or group-mating were kept at 28.5°C in Holtfreter’s solution (Kimmel et al., 1995). All animal experiments were performed in accordance with the guidelines approved by the Committee on Animal Experimentation of South China University of Technology (permission number 2022096).

### Plasmid, mRNA, MO and microinjection

The primitive vector *foxc1b:EosFP* was kindly provided by Professor Anming Meng (Tsinghua University, China). *foxc1b:pdgfαb-P2A-EosFP* plasmid was created with a fusion expression of *pdgfαb* and EosFP under the control of the *foxc1b* promoter (Qiu et al., 2016). Capped mRNAs were synthesized *in vitro* for Tol2 and caMEK1 from the corresponding linearized plasmids (Bolcome et al., 2010) using the mMessage mMachine SP6 kit (Ambion). Both plasmid and mRNA were injected into embryos at the one-cell stage. In the rescue experiments, 200 pg Tol2 mRNA and 30 pg *foxc1b:pdgfαb-P2A-EosFP* plasmids were co-injected into embryos. To constitutively activate ERK signaling, 20 pg caMEK1 mRNA was injected into embryos. To disrupt PDGF signaling, 7 ng *pdgfra* MO (5′-ATGGTCACGTAGATTGTGCTCAGCT-3′; Kartopawiro et al., 2014) and/or 10 ng *pdgfrb* MO (5′-ACAGGAACTGAAGTCACTGACCTTC-3′; Wiens et al., 2010) was injected into wild-type embryos.

### Whole-mount *in situ* hybridization

Digoxigenin-UTP-labeled antisense RNA probes for *gata1*, *hbbe3*, *npas4l*, *runx1*, *cmyb*, *pu.1*, *lcp1*, *scl*, *etv2*, *flk* (*kdrl*) (Liu et al., 2008), *pdgfαa*, *pdgfαb*, *pdgfra* and *pdgfrb* (Mao et al., 2019) were synthesized using a MEGAscript Kit (Ambion, USA) according to the manufacturer's instructions. WISH using these RNA probes was performed following standard procedures (Ning et al., 2013). The stained embryos were then processed to 10 µm thick cryosections. For dual-color fluorescence *in situ* hybridization, anti-digoxigenin-POD (1:400; 11633716001, Roche) and anti-fluorescein-POD (1:400; 11426346910, Roche) were used as primary antibodies to detect digoxigenin-labeled *pagfra* or *pdgfrb* probes and the fluorescein-labeled *gata1* probe, respectively, and the embryos were then stained with fluorescein tyramide (1:50; PerkinElmer) and cyanine 3 tyramide (1:50; PerkinElmer), respectively.

### Real-time quantitative PCR

Real-time quantitative PCR was performed as previously described (Yan et al., 2019). The primer sequences are listed in Table S1. The expression levels of *β-actin* was used as reference to normalize each sample.

### Immunostaining and western blotting

Embryos were harvested at the indicated stages, and immunostaining was performed as previously described (Ning et al., 2013). The following antibodies were used: anti-DsRed (1:200; 632496, Clontech, USA), anti-mCherry (1:200; ab125096, Abcam, UK), anti-p-PDGFRα (phospho Y849) (1:250; ab79318, Abcam, UK), anti-p-ERK1/2 (1:250; 9101, Cell Signaling Technology, USA), anti-p-AKT (1:400; 4060, Cell Signaling Technology, USA) and anti-BrdU (1:1000; B2531, Sigma, Japan). The secondary antibodies used were as follows: Alexa Fluor^®^ 488 AffiniPure donkey anti-rabbit IgG (1:200; 711-545-152, Jackson ImmunoResearch), Alexa Fluor^®^ 594 AffiniPure donkey anti-mouse IgG (1:200; 715-585-150, Jackson ImmunResearch), Alexa Fluor^®^ 488 AffiniPure donkey anti-mouse IgG (1:200; 715-545-150, Jackson ImmunoResearch) and Alexa Fluor^®^ 594 AffiniPure donkey anti-rabbit IgG (1:200; 711-585-152, Jackson ImmunoResearch). Anti-p-PDGFRα (phospho Y849) (1:1000; ab79318, Abcam, UK) and anti-β-actin (1:5000; 66009, Proteintech, USA) were used for western blotting experiments with donkey HRP-conjugated anti-rabbit IgG (1:10,000; NA934V, GE Healthcare) and sheep HRP-conjugated anti-mouse IgG (1:7000; NA931V, GE Healthcare) as the secondary antibodies, respectively.

### Confocal imaging

Confocal images of immunofluorescence or live embryos were captured using a Nikon A1R+ confocal microscope (20× or 10× objective), and the fluorescence intensity of immunofluorescence images was quantified using ImageJ version 1.48.

### Pharmacological treatment

Wild-type embryos were treated with 0.25 μM PDGFR tyrosine kinase inhibitor V (521234, Calbiochem, Germany) from shield stage to the indicated stages and then harvested for *in situ* hybridization. To block ERK1/2 signaling, embryos were exposed to 25 μM U0126 (U120-1MG, Sigma, Japan) from shield stage and harvested in the indicated stages for further experiments.

### *o*-Dianisidine staining

Embryos were harvested at 48 hpf. Staining of hemoglobin by *o*-dianisidine was performed as previously described (Ransom et al., 1996).

### Proliferation and apoptosis assays

Embryos at specific stages were subjected to 10 mM BrdU (B5002, Sigma) on ice for 20 min. The incorporated BrdU and DsRed protein were detected using anti-BrdU (1:1000; B2531, Sigma) and anti-DsRed (1:200; 632496, Clontech, USA), respectively, for whole-mount immunostaining. TUNEL assays were carried out using an *In Situ* Cell Death Detection Kit, TMR red (12156792910, Roche) following the manufacturer's instructions.

## Supplementary Material

10.1242/develop.201807_sup1 Supplementary information
