# Supplementary material for: Sclerotome-derived PDGF signaling functions as a niche cue responsible for primitive erythropoiesis
Source: Development. 2023 Nov 16;150(22):dev201807. doi: 10.1242/dev.201807 (PMC10690055; doi:10.1242/dev.201807)
Supplement: Supplementary information [file develop-150-201807-s1.pdf]

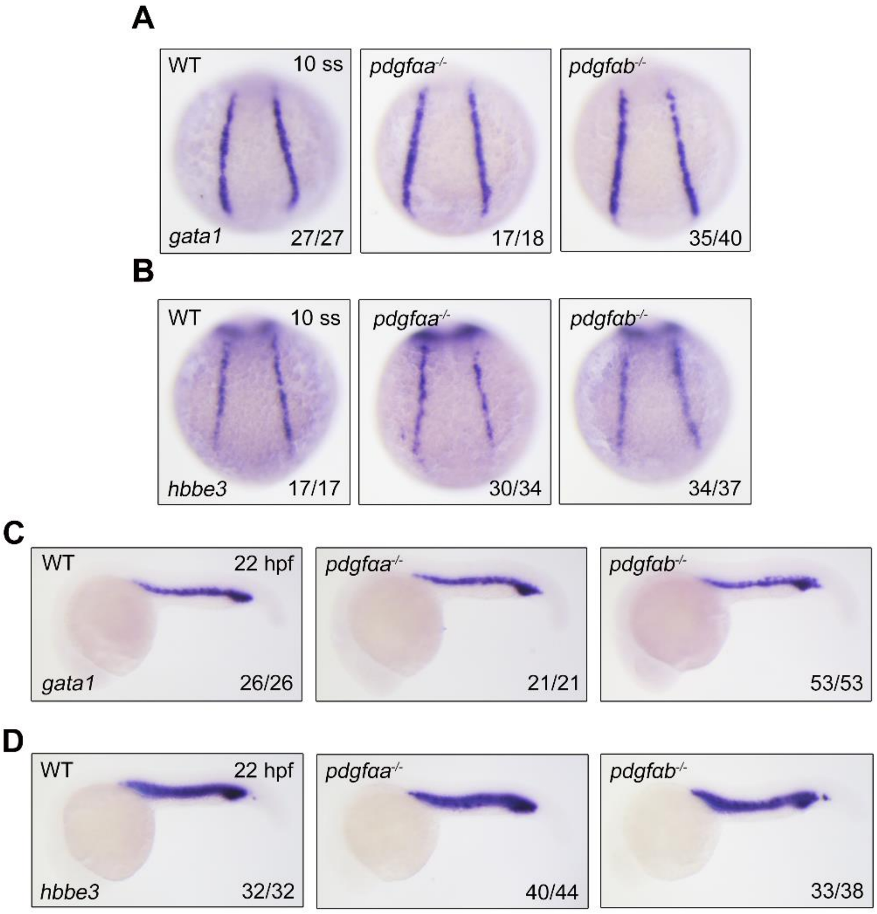

Fig. S1. The primitive erythropoiesis remains unaffected in *pdgfaa*$^{-/-}$ or *pdgfab*$^{-/-}$ mutants. (A,B) Expression analysis of *gata1* (A) and *hbbe3* (B) via *in situ* hybridization in *pdgfaa*$^{-/-}$ or *pdgfab*$^{-/-}$ mutants at the 10 somite stage. (C,D) Expression patterns of *gata1* (C) and *hbbe3* (D) at 22 hpf.

Development • Supplementary information

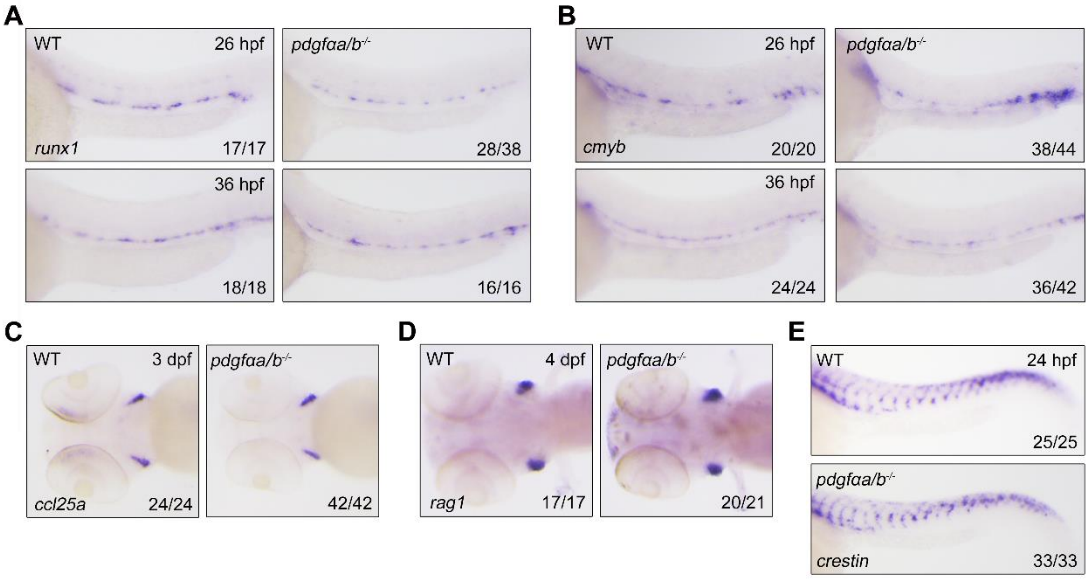

Fig. S2. Ablation of *pdgfaa* and *pdgfab* does not affect HSC formation. Expression patterns of *runx1*(A), *cmyb* (B), *ccl25a* (C), *rag1* (D), and *crestin* (E) in wild-type (WT) and *pdgfaa*$^{-/-}$;*pdgfab*$^{-/-}$ embryos at indicated stages.

Development • Supplementary information

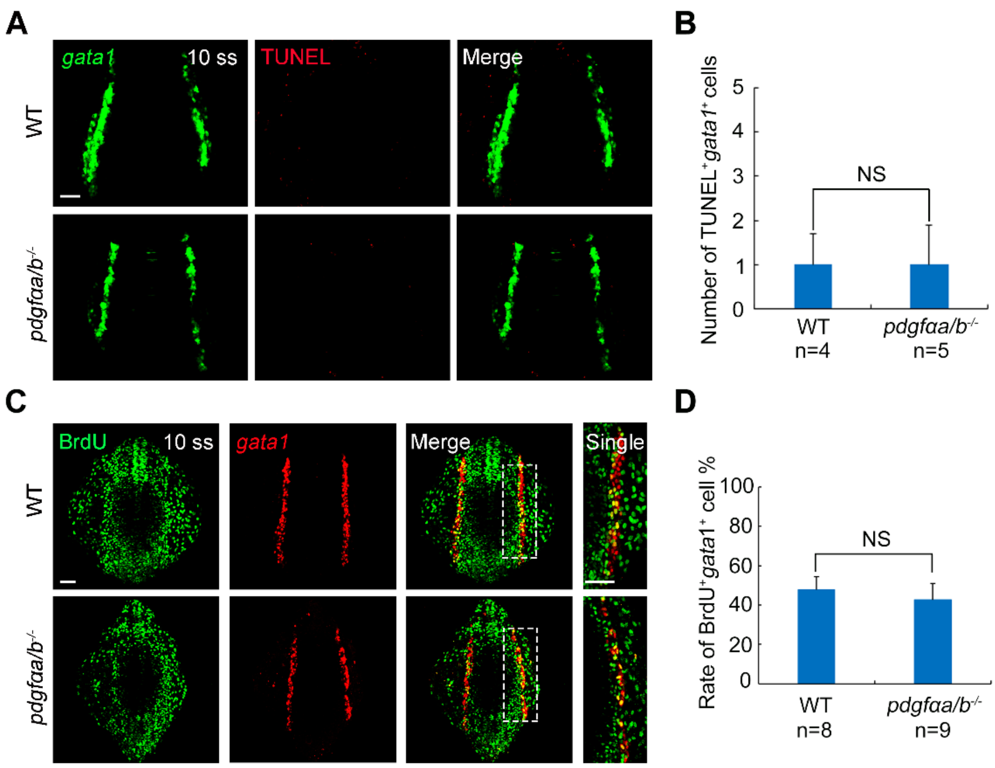

Fig. S3. Deletion of *pdgfaa* and *pdgfab* has no obvious effect on the apoptosis and proliferation of erythroid progenitors. (A,B) Cell apoptosis was detected by TUNEL assay in *Tg(gata1:dsRed)* embryos lacking *pdgfaa* and *pdgfab* (*pdgfaa/b*$^{-/-}$). Confocal images were shown in (A). Scale bar, 50 µm. The average numbers of apoptotic erythrocytes were quantified from three independent experiments and the group values were expressed as mean±SD. (B). Student's *t*-test. NS, no significance. (C,D) Cell proliferation was detected by BrdU staining in *Tg(gata1:dsRed)* embryos lacking *pdgfaa* and *pdgfab* (*pdgfaa/b*$^{-/-}$). Representative confocal images were displayed, with magnified views of the areas indicated by the dashed boxes (C). Scale bars, 50 µm. The average numbers of BrdU-positive erythrocytes were quantified from three independent experiments and the group values were expressed as mean±SD. (D). Student's *t*-test. NS, no significance.

Development • Supplementary information

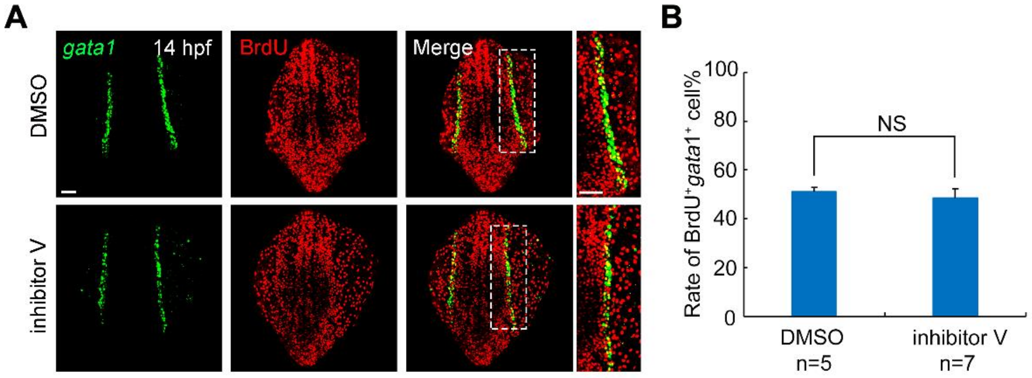

Fig. S4. Blocking PDGF signaling does not influence erythrocytic cell proliferation. (A,B) BrdU incorporation assays were performed in *Tg(gata1:dsRed)* embryos treated with DMSO or 0.25 µM inhibitor V from the shield stage to 14 hpf. Confocal images are shown in (A). The boxed areas were enlarged. Scale bar, 50 µm. The average numbers of BrdU-positive erythrocytes were quantified from three independent experiments and the group values were expressed as mean±SD. (D). Student's *t*-test. NS, no significance.

.

Development • Supplementary information

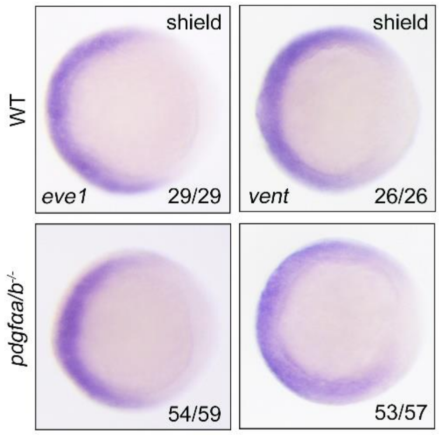

Fig. S5. Deletion of *pdgfαa* and *pdgfαb* does not affect the formation of ventral mesoderm. Expression analysis of *eve1* and *vent* in wild-type (WT) and *pdgfαa*$^{-/-}$;*pdgfαb*$^{-/-}$ (*pdgfαa/b*$^{-/-}$) embryos at the shield stage.

Development • Supplementary information

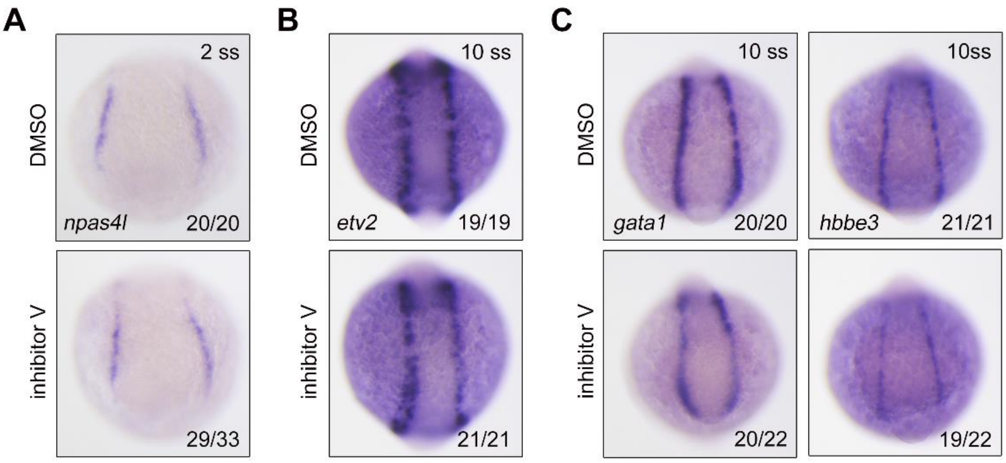

Fig. S6. Inhibition of PDGF signaling disrupts erythrocyte progenitor differentiation. (A) Expression pattern of *npas4l* in embryos treated with DMSO or inhibitor V. (B,C) Analysis of the expression of *etv2* (B) , *gata1* and *hbbe3* (C) in embryos treated with DMSO or inhibitor V.

Development • Supplementary information

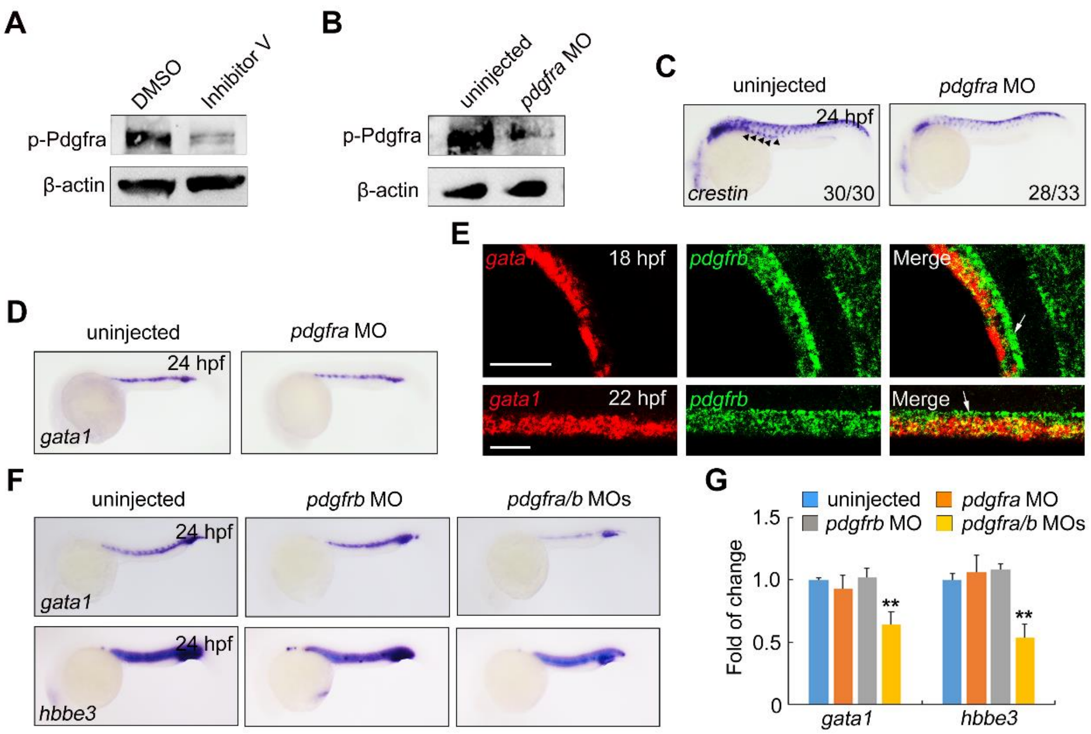

Fig. S7. Both *pdgfra* and *pdgfrb* are required for primitive erythropoiesis. (A,B) Embryos were treated with 0.25 µM inhibitor V (A) or injected with 7 ng *pdgfra* MO (B). The resulting embryos were harvested at 24 hpf and then subjected to western blot analysis with anti-p-Pdgfra antibody. (C,D) Analysis of the expression patterns of *crestin* (C) and *gata1* (D) in embryos injected with or without 7 ng *pdgfra* MO. (E) Co-localization analysis of *pdgfrb* and *gata1* in wild-type embryos at 18 hpf and 22 hpf by dual color fluorescence *in situ* hybridization. White arrow indicated the hypochord. Scale bar, 50 µm. (F,G) Embryos were injected with *pdgfrb* MO together with or without *pdgfra* MO at the 1 cell stage, and then harvested for expression analysis of *gata1* and *hbbe3* by in *situ* hybridization (F). Real-time quantitative PCR was performed to quantified the expression of *gata1* and *hbbe3* in the indicated embryos, and the values are shown as mean±SD from three independent experiments. Student's *t* test was used to analyze the statistical differences (G). ***P*<0.01.

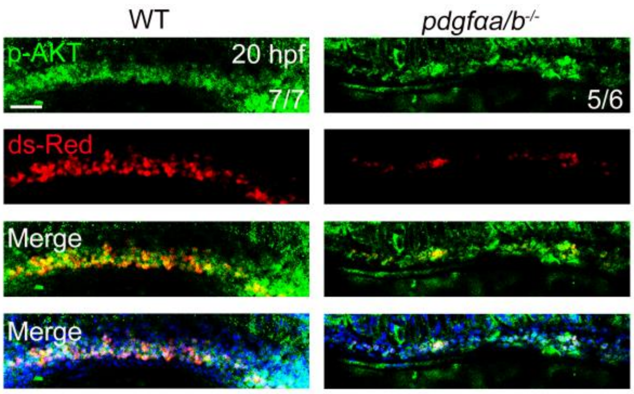

Fig. S8. Deletion of *pdgfαa* and *pdgfαb* does not obviously affect the expression level of p-AKT. Immunostaining of p-AKT in *Tg(gata1:dsRed)* embryos lacking *pdgfαa* and *pdgfαb* (*pdgfαa/b*$^{-/-}$) at 20 hpf. Scale bar, 50 µm.

Development • Supplementary information

Table S1. The primer sequences used to detect the expression of target genes.

**Gene Name**	Sequence (5’-3’)
hbae1-F	CTGACGACATCGGACACG
hbae1-R	CCTCCCATCACAGTCTTGC
hbbe3-F	GCAGCGATTCAGAACATC
hbbe3-R	CTTGAGGACCACAACACC
gata1-F	TCTGAGCCTTCTCGTTGG
gata1-R	CTGGACGCTGGTGGAATA
hbae3-F	GCCGTGAGACTCTTTCCAG
hbae3-R	TCCCTTCAGGTCATCCATC
hbbe2-F	GCAGAGGGCTTTGATTGTGTA
hbbe2-R	TCGGCGTAGGTGTTCTTGAT
β-actin-F	ATGGATGATGAAATTGCCGCAC
β-actin-R	ACCATCACCAGAGTCCATCACG

Development • Supplementary information

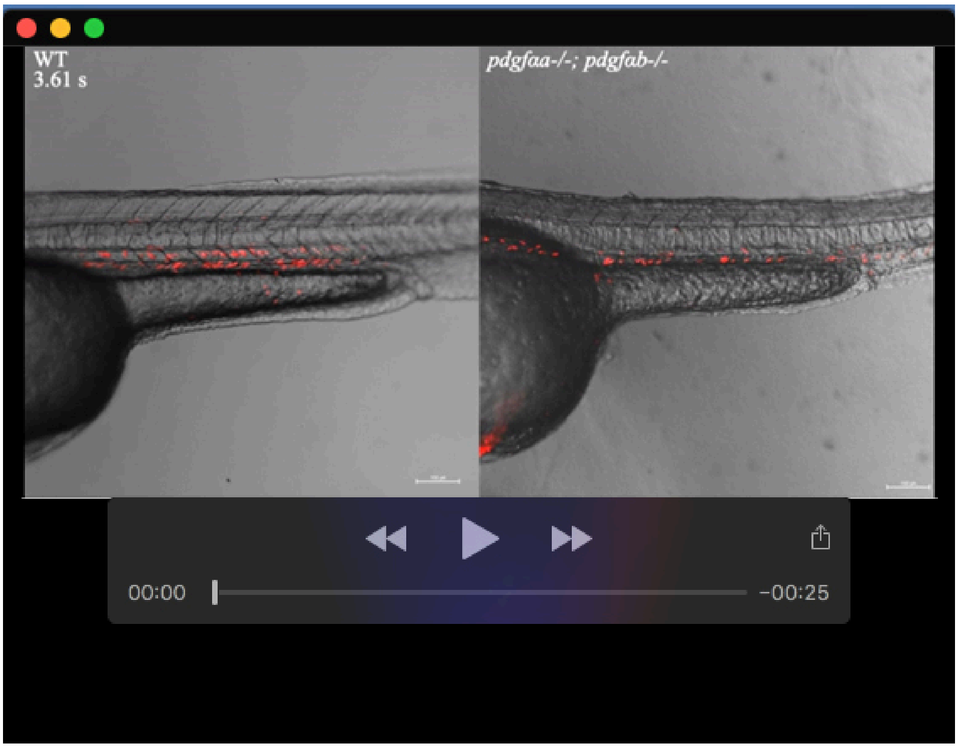

Movie 1. Confocal video shows a decreased number of erythrocytes in the blood flow of *Tg(gata1:dsRed)* embryos lacking *pdgfαa* and *pdgfαb* at 36 hpf.
