# Supplementary material for: Sclerotome-derived PDGF signaling functions as a niche cue responsible for primitive erythropoiesis
Source: Development. 2023 Nov 16;150(22):dev201807. doi: 10.1242/dev.201807 (PMC10690055; doi:10.1242/dev.201807)
Supplement: Supplementary file 1 [file develop-150-201807_review_history.pdf]

Sclerotome-derived PDGF signaling functions as a niche cue responsible for primitive erythropoiesis

Aihua Mao, Zhuyun Li, Guozhu Ning, Zhengrong Zhou, Chiju Wei, Jianchao Li, Xinyu He and Qiang Wang
DOI: 10.1242/dev.201807

Editor: Hanna Mikkola

Review timeline

Original submission:	23 March 2023
Editorial decision:	5 June 2023
First revision received:	22 September 2023
Accepted:	19 October 2023

Original submission

First decision letter

MS ID#: DEVELOP/2023/201807

MS TITLE: Sclerotome-derived PDGF signaling functions as a niche cue responsible for primitive erythropoiesis

AUTHORS: Aihua Mao, Zhuyun Li, Guozhu Ning, Zhengrong Zhou, Chiju Wei, Jianchao Li, Xinyu He, and Qiang Wang

I have now received all the referees' reports on the above manuscript, and have reached a decision. The referees' comments are appended below, or you can access them online: please go to BenchPress and click on the 'Manuscripts with Decisions' queue in the Author Area.

As you will see, the referees express considerable interest in your work, but have some significant criticisms and recommend a substantial revision of your manuscript before we can consider publication. If you are able to revise the manuscript along the lines suggested, which may involve further experiments, I will be happy receive a revised version of the manuscript. Your revised paper will be re-reviewed by one or more of the original referees, and acceptance of your manuscript will depend on your addressing satisfactorily the reviewers' major concerns. Please also note that Development will normally permit only one round of major revision. If it would be helpful, you are welcome to contact us to discuss your revision in greater detail. Please send us a point-by-point response indicating your plans for addressing the referee’s comments, and we will look over this and provide further guidance.

Please attend to all of the reviewers' comments and ensure that you clearly highlight all changes made in the revised manuscript. Please avoid using 'Tracked changes' in Word files as these are lost in PDF conversion. I should be grateful if you would also provide a point-by-point response detailing how you have dealt with the points raised by the reviewers in the 'Response to Reviewers' box. If you do not agree with any of their criticisms or suggestions please explain clearly why this is so.

Reviewer 1

Advance summary and potential significance to field

© 2023. Published by The Company of Biologists under the terms of the Creative Commons Attribution License (https://creativecommons.org/licenses/by/4.0/).

This manuscript provides genetic evidence for a role for PDGF signaling in primitive erythropoiesis in the zebrafish. The authors also show that the PDGF signal arises from cells of the sclerotome and affects primitive erythropoiesis but not myelopoiesis.

Comments for the author

The authors have examined primitive erythropoiesis (the first wave of erythropoiesis seen in the embryo) in pdgfαa-/-;pdgfαb-/- double mutant and pdgfαa-/- single mutant zebrafish embryos using o-dianisidine staining for hemoglobin, whole-mount in situ hybridization of the erythroid markers gata1 and hbbe3, a selective PDGFR tyrosine kinase inhibitor. Hemoglobin staining and expression of gata1 and hbbe3 were reduced in the double mutants. Single pdgfαa-/- mutants do not display a defect in primitive erythropoiesis, suggesting possible compensation by pdgfab-/-. In contrast, expression of several markers of definitive (second wave) hematopoiesis was not affected. Cell proliferation and survival were not affected in the double mutants. Analysis of ventral mesoderm and hemangioblast markers in the double null mutants or upon inhibition using the PDGFR inhibitor revealed no differences in these cell populations. Expression of 3 myeloid markers was also unaffected. Mapping of the temporal and spatial expression of pdgfαa and pdgfαb and their receptor pdgfra suggested that the PDGF signal arises from the sclerotome. Overexpression of pdgfab using a sclerotome specific gene promoter resulted in rescue of gata1 expression. Immunostaining experiments demonstrated that ERK1/2 but not AKT signaling is significantly reduced in the double mutants. Expression of a constitutively active form of MEK1 rescued expression of hemoglobin. Taken together, these findings support a role for PDGF signaling in primitive erythropoiesis in the zebrafish embryo.

The data presented in this manuscript support the conclusions. I am not sure that it is accurate to say that the sclerotome is the niche, however. The PDGF signal apparently arises in the sclerotome but those cells are adjacent to the mesoderm from which primitive erythroid progenitors arise.

The findings in zebrafish are quite different from those in the mouse (which suggest a role for PDGF-B signaling in the trophoblast cells of the placenta); these differences should be discussed.

Some minor comments:

Abstract: please clarify intended meaning of “consecutively resulting in…”
The first sentence of the Introduction needs to be revised: “the most common cell type in the blood responsible for oxygen transportation…” As written, the sentence implies that other cell types are also responsible for oxygen transportation.

RE vital role of primitive red blood cells: these cells also play a role in vascular development
Line 72-73: “having been widely investigated in recent decades” – I recommend revising to “and have been widely….”
Line 86: to my knowledge, hypereosinophilia is not a malignancy
Lines 90 and 233: the first reference is incomplete (“D et al., 2022)
Line 91: I recommend revising “remains largely elusive” to “is unknown” or something comparable
Line 93: “responsible for” is not an accurate description – “plays a role in” would be better.

Line 101-102: “To confirm this observation, o-dianisidine staining…” Staining reflects the amount of hemoglobin, not the numbers of cells. If the authors wish to indicate that staining facilitated detection of the cells, they should say so.

Figures C, D, F: What is the significance of the fractions given on each panel? For example, for 21/36, what do the remaining 15 samples show?

Line 133: “significantly” would be a better choice of words than “remarkablely” (which is also misspelled)

Line 136: “possible” would be a better choice of words than “potential”

Line 143-144: I don’t see a TUNEL signal (therefore it does not make sense to say “TUNEL staining revealed similar apoptotic signal…”)

© 2023. Published by The Company of Biologists under the terms of the Creative Commons Attribution License (https://creativecommons.org/licenses/by/4.0/).

Line 151: “vascular endothelial cell” should read “cells”

Line 152: “scrutinized” should be replaced with a more appropriate word, e.g. “evaluated”

Line 158-159: “in contrast” would be a better choice of words than “on the contrary”
Line 161: “Moreover” or “in addition” would be a better choice of words than “Besides”

Line 163: I am not sure what the authors mean by “Consistently” – do they mean they observed this in a large number of samples or that this observation is consistent with something else?

Line 204: I recommend rewording to “As PDGF receptors have been reported to activate….”

Line 207, sentence beginning “To shed light on this,…” I recommend deleting “To shed light on this”

Line 222: I recommend replacing “we could conclude” with “these findings indicate that” or something equivalent

Line 227: activating should read “activate”

Line 243: “as well” should be deleted

<u>Reviewer 2</u>

Advance summary and potential significance to field

The manuscript by Mao et al describes the role of PDGF signaling in zebrafish erythropoiesis. The authors show that zebrafish pdgfaa; pdgfab mutants display reduced erythropoiesis, which is correlated with reduced Pdgfra and ERK phosphorylation. Sclerotome specific expression of pdgfab was sufficient to partially rescue red blood cell differentiation in pdgfaa; pdgfab mutant embryos. In addition, activation of ERK signaling partially rescued hematopoieti defects in pdgfaa; pdgfab mutant embryos. Authors conclude that sclerotome-derived PDGF signaling promotes erythroid differentiation by inducing ERK signaling.

Overall, the study is clear, the manuscript is well written and the findings show a novel role for PDGF signaling in embryonic erythropoiesis, and provide a mechanistic insight into its function.

Comments for the author

Authors should consider addressing the following points.

1. Phenotyping analysis and rescue experiments are based mostly on in situ hybridization analysis of marker expression and subjective evaluation of marker reduction level. Such analysis can be biased and ISH approach is not well suited for quantitative evaluation for marker expression. It is recommended to confirm the key results in rescue experiments (Fig. 3H, Fig. 4G,H) by using qPCR which would provide better quantification of marker expression.

2. Is pdgfra expressed in blood progenitors? While it is apparent from the sections that pdgfra expression is present in the ICM region, it is difficult to tell which cells show pdgfra expression. Two color fluorescent ISH could answer this question.

3. It is difficult to tell which cells are positive for p-PDGFRa and p-ERK expression in Fig. 4A,B. Does the signal overlap in erythroid cells? Analysis of single confocal slices at higher magnification may help to answer this question. If the signal does not overlap, how does that affect the proposed model?

Also, can the authors clarify embryo orientation in this figure. What tissue is the green line of more dorsally located cells, positive for PDGFRa and p-ERK?

© 2023. Published by The Company of Biologists under the terms of the Creative Commons Attribution License (https://creativecommons.org/licenses/by/4.0/).

4. Can the authors note how many times the experiment in Fig. 4A,B has been replicated? Based on the embryo numbers, it appears that this analysis may have been performed only once. Quantification of staining intensity would be helpful.

Has p-PDGFRa antibody been previously validated in zebrafish? If not, then additional experiments may be needed to confirm its specificity.

5. Does pdgfab overexpression expand erythropoiesis? Would foxca1b:pdgfab overexpression in wild-type embryos result in expanded formation of red blood cells? It would be also very interesting to test if such overexpression of pdgfab could induce red blood cells in cloche / npas4l mutants if these mutatns were available to the authors.

6. Does foxca1b:pdgfab overexpression restore p-PDGFRa and p-ERK phosphorylation in pdgfaa, pdgfab mutants?

6. Erytrhopoietic defects have not been previously reported in pdgfra / pdgfrb mutants. In fact, PdgfraMO or Pdgfrb MO injection is reported to have no effect on gata1 expression or primitive blood formation (Damm and Clements, Nat Cell Biol 2017). It would be highly beneficial to test if genetic pdgfra / b mutants also have similar defects. Chemical inhibitors of Pdgfr signaling may have other non-specific defects. It has been reported that Pdgfra and Pdgfrb MOs could be used if mutants are unavailable, and p-PDGFR antibody could be a great tool to confirm MO efficacy.

First revision

Author response to reviewers' comments

Point-to-point responses to reviewers' concerns:

The major changes were highlighted with red color in the revised manuscript.

Reviewer 1
Advance Summary and Potential Significance to Field:
This manuscript provides genetic evidence for a role for PDGF signaling in primitive erythropoiesis in the zebrafish. The authors also show that the PDGF signal arises from cells of the sclerotome and affects primitive erythropoiesis but not myelopoiesis.

Reviewer 1 Comments for the Author:
The authors have examined primitive erythropoiesis (the first wave of erythropoiesis seen in the embryo) in *pdgfaa*$^{-/-}$;*pdgfab*$^{-/-}$ double mutant and *pdgfaa*$^{-/-}$ single mutant zebrafish embryos using o-dianisidine staining for hemoglobin, whole-mount in situ hybridization of the erythroid markers *gata1* and *hbbe3*, a selective PDGFR tyrosine kinase inhibitor. Hemoglobin staining and expression of *gata1* and *hbbe3* were reduced in the double mutants. Single *pdgfaa*$^{-/-}$ mutants do not display a defect in primitive erythropoiesis, suggesting possible compensation by *pdgfab*$^{-/-}$. In contrast, expression of several markers of definitive (second wave) hematopoiesis was not affected. Cell proliferation and survival were not affected in the double mutants. Analysis of ventral mesoderm and hemangioblast markers in the double null mutants or upon inhibition using the PDGFR inhibitor revealed no differences in these cell populations. Expression of 3 myeloid markers was also unaffected. Mapping of the temporal and spatial expression of *pdgfaa* and pdgfαb and their receptor *pdgfra* suggested that the PDGF signal arises from the sclerotome. Overexpression of *pdgfab* using a sclerotome specific gene promoter resulted in rescue of gata1 expression. Immunostaining experiments demonstrated that ERK1/2 but not AKT signaling is significantly reduced in the double mutants. Expression of a constitutively active form of MEK1 rescued expression of hemoglobin. Taken together, these findings support a role for PDGF signaling in primitive erythropoiesis in the zebrafish embryo.

© 2023. Published by The Company of Biologists under the terms of the Creative Commons Attribution License (https://creativecommons.org/licenses/by/4.0/).

The data presented in this manuscript support the conclusions. I am not sure that it is accurate to say that the sclerotome is the niche, however. The PDGF signal apparently arises in the sclerotome but those cells are adjacent to the mesoderm from which primitive erythroid progenitors arise.
Response: Thanks for these encourage comments. Niche was firstly proposed by Schofield in 1978, as a stereotyped microenvironment that protects the stem cells or progenitor cells and regulates their behavior (Scadden, 2006; Schofield, 1978). Moreover, the elements of niches including specific anatomic locations and structures, cell types, and extracellular signals interacted with the resident stem cell. These niche factors function to maintain the stem cells quiescent or induce them differentiation and self-renew. Our work indicates that sclerotome cells express PDGFs, which bind with PDGFRs enriched in the adjacent lateral plate mesoderm to support erythroid progenitor differentiation. Importantly, specifically overexpressed *pdgfab* in the sclerotomal cells could significantly restored the decreased expression of phosphorylated-PDGFRa and ERK in *pdgfaa*$^{-/-}$*;pdgfab*$^{-/-}$ double mutants, so as the defective differentiation of erythroid progenitors. These findings support the idea that sclerotome function as a hematopoietic niche responsible for primitive erythropoiesis.

The findings in zebrafish are quite different from those in the mouse (which suggest a role for PDGF-B signaling in the trophoblast cells of the placenta); these differences should be discussed.
Response: Thanks for pointing out this. We have discussed this issue as follows: Our findings strongly imply that sclerotome-derived PDGF signaling serves as a niche cue responsible for primitive hematopoiesis. Interestingly, it has been suggested that PDGFB signaling in the placental microenvironment plays a role in protecting hematopoietic stem/progenitor cells from definitive erythroid differentiation (Chhabra et al., 2012), implying that PDGF signaling might have distinct roles between primitive and definitive erythropoiesis.

Some minor comments:

Abstract: please clarify intended meaning of “consecutively resulting in…” The first sentence of the Introduction needs to be revised: “the most common cell type in the blood responsible for oxygen transportation…” As written, the sentence implies that other cell types are also responsible for oxygen transportation. RE vital role of primitive red blood cells: these cells also play a role in vascular development.
Response: Thanks for pointing out these issues. We have modified the relevant statement as follows:
1) Ablating *pdgfaa* and *pdgfab* or blocking PDGF signaling with an inhibitor impairs erythroid progenitor differentiation, thus resulting in a significant decrease in the number of erythrocytes.
2) Erythrocytes are the most common cell type in blood and are responsible for oxygen transportation and vascular morphogenesis during embryo development (Baron et al., 2012; Zhou et al., 2021).

Line 72-73: “having been widely investigated in recent decades” – I recommend revising to “and have been widely….”
Response: Thanks for the constructive suggestion. We have modified the relevant statement as follows: These dedicated niches have been widely investigated in recent decades and comprise vascular endothelial cells, mesenchymal stromal cells, along with diverse molecular signals.

Line 86: to my knowledge, hypereosinophilia is not a malignancy
Response: Thanks for pointing out this error. We have modified the related statement as follows: In addition, aberrant activation of PDGF signaling is associated with hematopoietic malignancies, such as myeloid neoplasms with hypereosinophilia and large granular lymphocytic leukemia.

Lines 90 and 233: the first reference is incomplete (“D et al., 2022)
Response: Thanks for pointing out this mistake. We have modified the mistake in the revised text by updating the citations.

Line 91: I recommend revising “remains largely elusive” to “is unknown” or something comparable
Line 93: “responsible for” is not an accurate description – “plays a role in” would be better.
Response: Thanks for these two suggestions. We agree with the review’s comments and the related statements have been modified in the revised text.

© 2023. Published by The Company of Biologists under the terms of the Creative Commons Attribution License (https://creativecommons.org/licenses/by/4.0/).

Line 101-102: “To confirm this observation, o-dianisidine staining…” Staining reflects the amount of hemoglobin, not the numbers of cells. If the authors wish to indicate that staining facilitated detection of the cells, they should say so.
Response: We are sorry for the indistinct statement. According to the reviewer’s suggestion, we have modified the relevant statement as follows: To confirm this observation, o-dianisidine staining was applied at 48 hours post-fertilization (hpf) to detect the formation of RBCs through evaluating hemoglobin levels.

Figures C, D, F: What is the significance of the fractions given on each panel? For example, for 21/36, what do the remaining 15 samples show?
Response: Thanks for pointing out this issue. In Figure 1C,D,F, The fractions given on each panel indicate the ratio of affected embryos with representative image. For example, in Figure 1C, loss of *pdgfaa* and *pdgfab* down-regulated the expression of erythrocyte-specific markers *gata1*, 21 of the 36 embryos showed moderate decreased expression of *gata1*, and the remaining 15 embryos displayed severely reduced expression of *gata1*. We have modified the relevant statement as follows in the revised manuscript: After deletion of *pdgfaa* and *pdgfab*, the expression of erythrocyte-specific markers *gata1* and *hbbe3* was slightly decreased in about 60% of embryos and markedly reduced in about 40% of embryos at 22 hpf.

Line 133: “significantly” would be a better choice of words than “remarkablely” (which is also misspelled)
Line 136: “possible” would be a better choice of words than “potential”
Response: We are sorry for the spelling mistake and thanks for the suggestions. We have modified the relevant statements in the revised text.

Line 143-144: I don’t see a TUNEL signal (therefore it does not make sense to say “TUNEL staining revealed similar apoptotic signal…”)
Response: We are sorry for the inappropriate statement. We have modified the related statement as follows: TUNEL staining revealed that no obvious apoptotic signal was detected in the erythrocyte progenitor cells of wild-type and *pdgfaa*$^{-/-}$;*pdgfab*$^{-/-}$ embryos.

Line 151: “vascular endothelial cell” should read “cells”
Response: Thanks for pointing out this error. We have modified the related statement as follows: Because hemangioblasts arise out of ventral mesoderm and in turn differentiate into hematopoietic cells and vascular endothelial cells.

Line 152: “scrutinized” should be replaced with a more appropriate word, e.g. “evaluated”
Response: Thanks for pointing out this issue. We have modified the relevant statement in the revised text.

Line 158-159: “in contrast” would be a better choice of words than “on the contrary”
Line 161: “Moreover” or “in addition” would be a better choice of words than “Besides”
Response: Thanks for these suggestions. We have modified the relevant statements in the revised manuscript.

Line 163: I am not sure what the authors mean by “Consistently” – do they mean they observed this in a large number of samples or that this observation is consistent with something else?
Response: We are sorry for the indistinct statement. We have modified the sentence as follows: Consistent with the above observations, suppressing PDGF signaling with inhibitor V did not alter the formation of hemangioblast and the development of endothelial progenitors, but severely reduced the emergence of erythrocyte progenitors.

Line 204: I recommend rewording to “As PDGF receptors have been reported to activate….”
Response: Thanks for the suggestion. We have reworded the relevant sentence as follows: As PDGF receptors have been reported to activate several downstream signaling cascades including AKT-PI3K and ERK1/2 (Heldin, 2013; Hoch and Soriano, 2003), we sought to determine which pathway downstream of PDGF receptors plays a primary role in primitive erythropoiesis.

Line 207, sentence beginning “To shed light on this,…” I recommend deleting “To shed light on this”

© 2023. Published by The Company of Biologists under the terms of the Creative Commons Attribution License (https://creativecommons.org/licenses/by/4.0/).

Response: Thanks for the suggestion. We have deleted the relevant statement in the revised text.

Line 222: I recommend replacing “we could conclude” with “these findings indicate that” or something equivalent
Response: Thanks for the suggestion. We have revised the relevant statement as follows: Taken together, these findings indicate that ERK1/2 signaling acts downstream of PDGF signaling to regulate primitive erythropoiesis.

Line 227: activating should read “activate”
Response: Thanks for the pointing out this issue. We have modified the relevant statement in the revised manuscript.

Line 243: “as well” should be deleted
Response: Thanks for the pointing out this mistake. “as well” has been deleted in the related sentence in the revised manuscript.

Reviewer 2
Advance Summary and Potential Significance to Field:
The manuscript by Mao et al describes the role of PDGF signaling in zebrafish erythropoiesis. The authors show that zebrafish pdgfaa; pdgfab mutants display reduced erythropoiesis, which is correlated with reduced Pdgfra and ERK phosphorylation. Sclerotome specific expression of pdgfab was sufficient to partially rescue red blood cell differentiation in pdgfaa; pdgfab mutant embryos. In addition, activation of ERK signaling partially rescued hematopoieti defects in pdgfaa; pdgfab mutant embryos. Authors conclude that sclerotome-derived PDGF signaling promotes erythroid differentiation by inducing ERK signaling.
Overall, the study is clear, the manuscript is well written and the findings show a novel role for PDGF signaling in embryonic erythropoiesis, and provide a mechanistic insight into its function.
Response: Thanks for these encouraging comments.

Reviewer 2 Comments for the Author:
Authors should consider addressing the following points.
1. Phenotyping analysis and rescue experiments are based mostly on in situ hybridization analysis of marker expression and subjective evaluation of marker reduction level. Such analysis can be biased and ISH approach is not well suited for quantitative evaluation for marker expression. It is recommended to confirm the key results in rescue experiments (Fig. 3H, Fig. 4G,H) by using qPCR which would provide better quantification of marker expression.
Response: That’s a good point. Both the *in situ* hybridization and real-time quantitative PCR (qPCR) have been performed to evaluate the marker gene expression of erythroid progenitor in the revised manuscript. These two approaches showed similar results (Fig. 3I-K, Fig4G-I in the revised manuscript). For evaluating the amount of hemoglobin, *o*-dianisidine staining and qPCR for examining the expression of the hemoglobin genes have been performed (Fig. 4J-L in the revised manuscript). These results indicate that overexpression of *pdgfab* in the sclerotomal cells or overexpression caMEK1 could significantly restore the defective primitive erythropoiesis in the *pdgfaa*$^{-/-}$;*pdgfab*$^{-/-}$ mutants.

2. Is pdgfra expressed in blood progenitors? While it is apparent from the sections that pdgfra expression is present in the ICM region, it is difficult to tell which cells show pdgfra expression. Two color fluorescent ISH could answer this question.
Response: Thanks for the constructive suggestion. Dual color fluorescent *in situ* hybridization has been performed with *gata1*-Flu and *pdgfra*-Dig probes in the 14 hpf and 18 hpf embryos. Confocal image showed that *pdgfra* was specifically expressed in the erythrocyte progenitor cells expressing *gata1* during somitogenesis (Fig. 3F,G in the revised manuscript).

3. It is difficult to tell which cells are positive for p-PDGFRa and p-ERK expression in Fig. 4A,B. Does the signal overlap in erythroid cells? Analysis of single confocal slices at higher magnification may help to answer this question. If the signal does not overlap, how does that affect the proposed model?
Response: Thanks for pointing out this issue. To better examine which cells are positive for p-PDGFRa, we reperformed the immunostaining experiments using a p-PDGFR antibody.

© 2023. Published by The Company of Biologists under the terms of the Creative Commons Attribution License (https://creativecommons.org/licenses/by/4.0/).

Representative stack of confocal slices and single confocal slices with higher magnification have been provided in Fig. 3L in the revised manuscript. Furthermore, the single confocal slices have been enlarged in Fig. 4A in the revised manuscript. These results revealed that most of the p-PDGFRa$^+$ or p-ERK$^+$ cells in the ICM were erythroid cells. Importantly, ablation of *pdgfaa* and *pdgfab* significantly downregulated the phosphorylation level of PDGFRa or ERK in erythrocyte progenitors.

Also, can the authors clarify embryo orientation in this figure. What tissue is the green line of more dorsally located cells, positive for PDGFRa and p-ERK?

The embryo orientations in Fig. 3L and Fig. 4A in the revised manuscript have been indicated in the related Figure legends. For detail, please to see the revised text.

To identify the cell types of p-PDGFRa$^+$ and p-ERK$^+$ cell located in the more dorsally from the ICM, immunostaining was performed in the *Tg(rcn3:EGFP)* and *Tg(flk:GFP)* embryos individually. In the *Tg(rcn3:EGFP)* embryos, the signal of p-Pdgfra is located in the boundary of notochord which labeled by GFP. It indicated that the p-Pdgfra$^+$ green line located in the more dorsally is hypochord (See Figure below). The p-ERK$^+$ cells indicate by white arrows overlapped with the *flk*$^+$ arterial endothelial cells. These results indicate that the more dorsally p-ERK$^+$ cells are endothelial cells of the dorsal aorta. Due to the lack of correlation between the above results and our research, this result was not described in the revised manuscript.

NOTE: We have removed unpublished data that had been provided for the referees in confidence.

Expression analysis of p-Pdgfra in the *Tg(rcn3:EGFP)* embryos (A) and p-ERK in the *Tg(flk:GFP)* embryos (B) at 20 hpf by immunostaining. Scale bar, 20 μm. White arrows in (A) and (B) indicate the hypochord and dorsal aorta, respectively.

4. Can the authors note how many times the experiment in Fig. 4A,B has been replicated? Based on the embryo numbers, it appears that this analysis may have been performed only once. Quantification of staining intensity would be helpful.
Response: Thanks for pointing out this issue. The immunostaining assays in Fig.4A,B were repeated independently three times with similar results. Only the numbers of embryo from one of these experiments were shown in the manuscript. According to the suggestions from the reviewer, the average fluorescent intensity of p-PDGFRa and p-ERK in ICM region was quantified by software Image J and the values were shown as mean ± SD Student's *t*-test was used to analyze the statistic difference between wild-type and *pdgfaa*$^{-/-}$;*pdgfab*$^{-/-}$ embryos (Fig. 3M and Fig. 4B in the revised manuscript). These results support the idea that loss of *pdgfaa* and *pdgfab* significantly down-regulated the expression of p-PDGFRa and p-ERK.

Has p-PDGFRa antibody been previously validated in zebrafish? If not, then additional experiments may be needed to confirm its specificity.
Response: That's a good point. We evaluated the antibody while the results didn't show at that time. WT embryos were exposed to DMSO or 0.25 μm inhibitor V from shield stage, then harvested at 24 hpf for western blotting analysis with the p-PDGFRa antibody. Compared with the control group, blocking of PDGF signaling by inhibitor V resulted of remarkably down-regulated p-PDGRa (Fig.S7A in the revised manuscript). This result demonstrates the specificity of the p-PDGRa antibody used in our study.

5. Does pdgfab overexpression expand erythropoiesis? Would foxca1b:pdgfab overexpression in wild-type embryos result in expanded formation of red blood cells? It would be also very interesting to test if such overexpression of pdgfab could induce red blood cells in cloche / npas4l mutants if these mutatns were available to the authors.
Response: Thanks for these suggestions. In order to examine whether *pdgfab* overexpression would expand erythropoiesis, the plasmid *foxc1b:pdgfab* and tol2 mRNA were co-injected into wild-type embryos. However, the expression of *gata1* exhibited no changed when compared to control embryos (See Figure below, panel A). This may be due to that the endogenous expression of *pdgfab* in the sclerotome is sufficient for erythropoiesis or the function of PDGF signaling in erythropoiesis requires other unknown factors. Furthermore, no *gata1* transcripts were detected after overexpression *pdgfab* in the sclerotome of *cloche*$^{-/-}$ embryos (See Figure below, panel B), suggesting that the PDGF signaling dose not act at the downstream of *cloche* gene to promote erythropoiesis. These negative results were not described in the revised manuscript.

© 2023. Published by The Company of Biologists under the terms of the Creative Commons Attribution License (https://creativecommons.org/licenses/by/4.0/).

NOTE: We have removed unpublished data that had been provided for the referees in confidence.

Expression analysis of *gata1* in the indicated embryos by *in situ* hybridization.

6. Does foxca1b:pdgfab overexpression restore p-PDGFRa and p-ERK phosphorylation in pdgfaa, pdgfab mutants?
Response: This is a good point. Immunostaining staining experiments for detecting p-PDGFRa or p-ERK was performed in *pdgfaa*$^{-/-}$;*pdgfab*$^{-/-}$ embryos injected with or without *foxc1b-pdgfab-p2a-EosFP* plasmids and Tol2 transposase mRNA, then the average fluorescent intensity of the indicated protein in the ICM region was quantified. These results showed that overexpression of *pdgfab* in the sclerotome could significantly recover the expression of p-PDGFRa and p-ERK (Fig.3L,M and Fig.4E,F in the revised manuscript).

7. Erytrhopoietic defects have not been previously reported in pdgfra / pdgfrb mutants. In fact, PdgfraMO or Pdgfrb MO injection is reported to have no effect on gata1 expression or primitive blood formation (Damm and Clements, Nat Cell Biol 2017). It would be highly beneficial to test if genetic pdgfra / b mutants also have similar defects. Chemical inhibitors of Pdgfr signaling may have other non-specific defects. It has been reported that Pdgfra and Pdgfrb MOs could be used if mutants are unavailable, and p-PDGFR antibody could be a great tool to confirm MO efficacy.
Response: Thanks for these suggestions. It has been reported that *pdgfra* MO injected embryos displayed normal primitive erythropoiesis (Damm and Clements, 2017). We further confirmed the efficiency of the morpholino that has been used to knockdown *pdgfra* expression (Damm and Clements, 2017). Indeed, injection of *pdgfra* MO into wild-type embryos markedly decreased the expression level of phosphorylated-PDGFRa and caused clear defects in the ventromedial migration of trunk neural crest cells (Fig. S7B,C in the revised manuscript). However, the expression of *gata1* in the ICM region of *pdgfra* morphants was not obviously changed (Fig. S7D,G in the revised manuscript). In general, PDGFRα and PDGFRβ proteins form homo- or heterodimers to be activated by PDGF ligands (Kazlauskas, 2017; Shim et al., 2010). Interestingly, we found that *pdgfrb* was expressed in the hypochord and in the erythroid progenitor cells located near its ventral region (Fig. S7E in the revised manuscript), indicating that *pdgfrb* may be relevant to erythropoiesis. We then injected a previously validated antisense MO targeting *pdgfrb* into wild-type embryos (Wiens et al., 2010). We found that although *pdgfrb* single morphants showed no defect in primitive erythropoiesis, *pdgfra* and *pdgfrb* double morphants exhibited significantly reduced expression of *gata1* and *hbbe3* (Fig. S7F,G in the revised manuscript). These data indicate that *pdgfra* and *pdgfrb* functions together to promote primitive erythropoiesis, similar to their role in regulating craniofacial development (Fantauzzo and Soriano, 2016).

Second decision letter

MS ID#: DEVELOP/2023/201807

MS TITLE: Sclerotome-derived PDGF signaling functions as a niche cue responsible for primitive erythropoiesis

AUTHORS: Aihua Mao, Zhuyun Li, Guozhu Ning, Zhengrong Zhou, Chiju Wei, Jianchao Li, Xinyu He, and Qiang Wang
ARTICLE TYPE: Research Report

I am happy to tell you that your manuscript has been accepted for publication in Development, pending our standard ethics checks.

Reviewer 1

Advance summary and potential significance to field

© 2023. Published by The Company of Biologists under the terms of the Creative Commons Attribution License (https://creativecommons.org/licenses/by/4.0/).

This manuscript provides genetic evidence for a role for PDGF signaling in primitive erythropoiesis in the zebrafish. The authors also show that the PDGF signal arises from cells of the sclerotome and affects primitive erythropoiesis but not myelopoiesis.

Comments for the author

The authors have responded appropriately to the previous review. However, I continue to disagree with referring to the sclerotome, the source of the PDGF signal, as a niche. Based on my own reading of the literature, especially regarding hematopoiesis, a niche refers to a tissue that contains multiple cell types, not adjacent tissue types. I think it would be fine to leave this as is and let the readers come to their own conclusions about this point.

Reviewer 2

Advance summary and potential significance to field

The manuscript by Mao et al describes the role of PDGF signaling in zebrafish erythropoiesis. The authors show that zebrafish pdgfaa; pdgfab mutants display reduced erythropoiesis, which is correlated with reduced Pdgfra and ERK phosphorylation. Sclerotome specific expression of pdgfab was sufficient to partially rescue red blood cell differentiation in pdgfaa; pdgfab mutant embryos. In addition, activation of ERK signaling partially rescued hematopoieti defects in pdgfaa; pdgfab mutant embryos. Authors conclude that sclerotome-derived PDGF signaling promotes erythroid differentiation by inducing ERK signaling. Overall, the study is clear, the manuscript is well written and the findings show a novel role for PDGF signaling in embryonic erythropoiesis, and provide a mechanistic insight into its function.

In the revision the authors have made an excellent job to address all the points made by the reviewers. The revised manuscript has been greatly improved.

Comments for the author

I have no further suggestions.

© 2023. Published by The Company of Biologists under the terms of the Creative Commons Attribution License (https://creativecommons.org/licenses/by/4.0/).
